# The association between single nucleotide polymorphism in interleukin-27 gene and recurrent pregnancy loss in Iranian women

**Published:** 2015-04

**Authors:** Zeinab Nematollahi, Hossein Hadinedoushan, Abbas Aflatoonian, Gilda Eslami, Nasrin Ghasemi

**Affiliations:** 1 *Department of Immunology, Reproductive Immunology Research Center, Shahid Sadoughi University of Medical Sciences, Yazd, Iran.*; 2 *Research and Clinical Center for Infertility, Shahid Sadoughi University of Medical Sciences, Yazd, Iran.*; 3 *Department of Parasitology and Mycology, Faculty of Medicine, Shahid Sadoughi University of Medical Sciences, Yazd, Iran.*

**Keywords:** *Cytokine*, *IL*-*27*, *Inflammation*, *Polymorphism*, *Recurrent abortion*

## Abstract

**Background:**

Recurrent pregnancy loss (RPL) has been defined as two or more miscarriages before 20^th^ week of gestation. It seems that IL-27 may reduce inflammatory responses and affect the survival of the embryo during human pregnancy. *IL-27* polymorphisms may influence RPL by altering the levels or the activity of gene product.

**Objective:**

We studied for the first time the association of *IL-27* -964 A>G single nucleotide polymorphism (SNP) with RPL in Iranian women.

**Materials and Methods:**

A case-controlled study was performed on two groups consisting of 150 healthy women with at least one delivery (control group) and 150 women with two or more primary RPLs history (RPL group). The -964 A>G SNP in *IL-27* gene was determined by PCR-RFLP technique. Genotype and allele frequencies were compared using ^2^ tests between two groups.

**Results:**

There was no difference between the two groups regarding age of women (29±4.4 [control] vs. 30.84±5.2 years [case]). In the RPL group, the genotype frequencies of -964 A>G polymorphism were AG (49.3%), AA (40%), and GG (10.7%), and in the control group, they were AG (43.3%), AA (48.7%), and GG (8%). There was no significant difference between the genotypes of AA, AG, and GG in two groups (p=0.23). As the frequency of allele A was 64.7% in the RPL group and 70.3% in the control group, the difference in frequency of allele A in -964 A>G between two groups was not significant (p=0.19).

**Conclusion:**

Our findings indicate that SNP of -964 A>G in *IL-27* gene is not a risk factor for RPL in Iranian women.

## Introduction

Recurrent pregnancy loss (RPL) is defined as the occurrence of two or more miscarriages before the 20^th^ week of gestation that affects up to 3% of women of gestational age (1). RPL is still a major problem worldwide (2). While many of etiological factors in RPL such as chromosomal abnormalities, endocrine and anatomical disorders and infections have been well-established, in nearly half of the cases, etiology is still unknown (3). The role of some immunological factors in RPL was studied but further studies are needed to identify relationship between successful pregnancy and immune system (4). During human pregnancy, a semi-allogeneic fetus transplants in the uterus. In the normal pregnancy at the feto-maternal line some grades of systemic or local inflammation are necessary. Excessive inflammation might cause pregnancy complications such as RPL (5, 6).

 Recently a novel family of T helper (Th) cells was identified which was known by IL (Interleukin)-17 production and named Th17. IL-17, a pro-inflammatory cytokine, induces the production of many mediators of inflammation (7, 3). This cytokine may play a role in the maintenance of fetus during pregnancy. Wang *et al* found that the proportion of Th17 cells in the peripheral blood and the decidua of women with inevitable abortion were significantly higher than women with normal pregnancy (8). IL- 17F gene polymorphism might be considered as a risk factor for RPL in Iranian women (9).

IL-27 has been determined to be a new heterodimer cytokine of the IL-12 family that consists of IL-12p40-related protein encoded by the Epstein-Barr virus-induced gene 3 and IL-12 p35-related polypeptide encoded by the *p28* gene. Recent studies have revealed that IL-27 inhibits differentiation of Th17 cells (10, 11). It has been shown that IL-27 suppresses IL-17 production by activated CD4^+^ T cells. The inhibitory effect of IL-27 was also well noticeable on IL-23, another IL-12 related- cytokine with proinflammatory effects (12). Sasaoka *et al* found that treatment with IL-27 attenuates experimental colitis through the suppression of the development of IL-17- producing T helper cells (13). This cytokine inhibits excess proangiogenic activity, suppresses T-cell responses or controls inflammatory responses in normal pregnancy (14). 

The human *IL-27* gene is located on chromosome 16p11 and consisted of five exons. This gene is one of the crucial candidate genes for the development and differentiation of T cells (15). Associations of IL-27 gene polymorphisms with susceptibility to inflammatory diseases, autoimmune diseases and malignancy have been reported (16-18). Although It seems that IL-27 may reduce inflammatory responses through human pregnancy, IL-27 -964 A>G polymorphism is a genetic marker for identifying women at increased risk of recurrent spontaneous abortion. 

We hypothesized that this cytokine could affect the survival of the embryo during pregnancy and that IL-27 polymorphisms may influence RPL by altering the levels or the activity of gene product. To date no studies have investigated the association between IL- 27 gene polymorphism and RPL. This research was designed to study the association of IL-27p28 (-964 A/G) gene single nucleotide polymorphism (SNP) with RPL in Iranian women.

## Materials and methods

**Participants**

This study was carried out as a case- control study on two different groups. The control group consisted of 150 healthy women in reproductive age with no history of abortion and at least one successful pregnancy. The case group (PRL group) consisted of 150 women with two or more primary RPLs history. The case samples included women referred to Research and Clinical Center for Infertility, Yazd, Iran from March 2010 to September 2013. Our inclusion criteria were primary RPL (without any previous viable pregnancies) with no male factor cause nor anatomical and endocrinal abnormality, normal parental karyotyping, TORCH syndrome-negative, the absence of anti-phospholipid antibodies (IgM and IgG classes), anti-cardiolipin antibodies (IgM, IgG and IgA classes), and anti-nuclear antibodies (IgM and IgG classes). We also checked serum thyroid hormones, anti-thyroid peroxidase and anti-thyroglobulin and those thyroid disorders were excluded from the study. Ethics Committee of Shahid Sadoughi University of Medical Sciences, Yazd, Iran approved the study. Informed written consent was obtained from all of the enrolled participants. Healthy subjects as well as information for variables such as age, number of abortions, and number of pregnancies was provided for further analysis. 5 ml peripheral blood samples were taken from all participants and were collected in EDTA-treated tubes for DNA extraction. 

**Genotyping of IL-27** **-** **964 A>G**

Genomic DNA was extracted from whole blood samples using a DNA isolation kit according to the manufacturer's instructions (Bioneer, Republic of Korea) and stored at -20^o^C until needed. The genotyping of -964 A>G in IL-27 gene was performed using polymerase chain reaction restriction fragment length polymorphism (PCR-RFLP) method. Forward and reverse primers were designed according to the published sequences in the Genome Database (GenBank) using the PRIMER3 software. Information on primer sequences is as follows:

Forward: 5΄-AACTAGGGTCAGGGCTGGAT-3’

Reverse: 5΄-CCTCGGCAGATGTTGGTATT-3’

The PCR reaction was done in a 25 μl reaction volume containing 4 μl of DNA, 10 μl master mix (Ampliqon, Denmark), 8.5 μl distilled water and 1.25 μl of each specific reverse and forward primers. The PCR protocol was performed with an initial denaturation at 94^o^C for 5 min, followed by 30 cycles of denaturation for 1 min at 94^o^C, annealing for 45 sec at 62^o^C and extension for 90 sec at 72^o^C. Final extension was carried out for 5 min at 72^o^C (Applied Biosystems, ABI, Foster City, CA, USA). The product size of PCR was 753 base pairs (bp) confirmed with electrophoresis in a 1% agarose gel stained with DNA green viewer (Pars-Tous, Iran). 

The PCR product was digested by addition of restriction enzyme Ava1 (Fermentase, CA) at 37^ο^C for 60 min in a final volume of 30.2 µl under the following conditions; 10 μl PCR product, 2 μl buffer, 18 μl nuclease free water and 0.2 μl Ava 1. This enzyme recognizes nucleotide sequences 5’…C↓Y C G R G…3’ and cut it to the desired location. The resulting fragments of the RFLP were analyzed using electrophoresis on 2% gel agarose stained with DNA green viewer. The gels were imaged using an E-gel imager (Life Technologies). About 10% of the samples were randomly selected and the assays were repeated and the results were 100% consistent with the initial analyses. SNPs were evaluated for deviation from Hardy-Weinberg Equilibrium for both RPL and control groups. 

**Statistical analysis**

Statistical analysis was performed with SPSS version 16 (SPSS Inc, Chicago, IL, USA). SNPs were evaluated for deviation from Hardy-Weinberg Equilibrium by Chi-square test. The genotype and allele frequencies of SNP -964 A>G were calculated by direct count. The differences in allele and genotypes frequencies between women with RPL and controls were determined using Chi-square test. P<0.05 were considered statistically significant. The data are presented as mean±SD.

## Results

**Figure 1:**
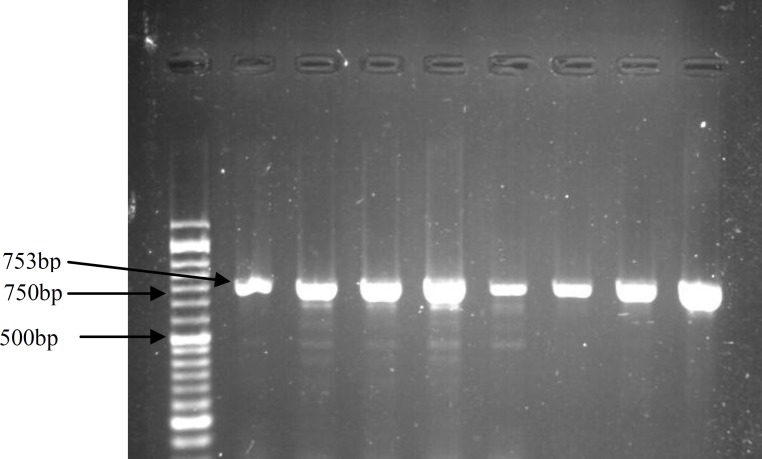
Gel picture showing PCR product for SNP -964 A>G. The PCR product size was 753bp.

**Figure 2:**
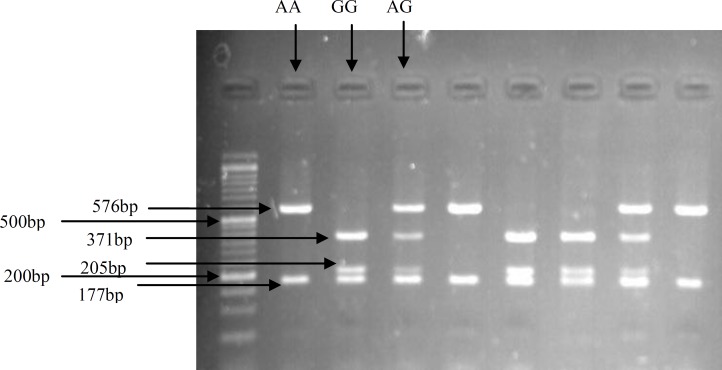
Gel picture showing RFLP fragments for SNP -964 A>G. Enzymatic digestion for the GG genotype generated 177, 205 and 371 bp fragments; 177, 205, 371 and 576 bp fragments for AG genotype, and 177 and 576 bp for AA genotype

The mean age of the women in control group was 29±4.4 years (range 20-41) and 30.84±5.2 years (range 22-42) in case group. There was no significant difference in the age of woman in the two groups. Characteristics of the RPL and control groups are shown in table I. All genotypes of the polymorphism were in Hardy-Weinberg Equilibrium. The undigested PCR product size was 753bp for SNP -964 A>G (Figure 1). Restriction digestion for the GG genotype generated 177, 205 and 371 bp fragments; whereas the AG genotype generated 177, 205, 371 and 576 bp fragments. Also, there were two bands (177 and 576 bp) in the presence of homozygous AA (Figure 2). 

The frequencies of genotypes of this polymorphism in the case group were AG (49.3%), AA (40%) and GG (10.7%), Moreover; they were AG (43.3%), AA (48.7%) and GG (8%) in the control group. There was no significant difference in genotype frequencies of the -964 A>G polymorphism between case and control groups (p=0.23). The allele frequencies of this polymorphism were A (64.7%), G (35.3%) in the RPL group and A (70.3%), G (29.7%) in the control group. The difference in frequencies of A and G alleles in the two groups were not significant (p=0.19) (Table II).

**Table I:** Characteristics of the RPL and control group

	**RPL Group**	**Control Group**
Age (yr)	30.84 ± 5.2 (22-42)	29 ± 4.4 (20-41)
No. of RPL	3 ± 1.3 (2-7)	0
No. of pregnancy	0	2.3 ± 0.92(2-5)

Data are presented as Mean±SD (Range).

RPL= Recurrent pregnancy loss

**Table II:** The frequencies of *IL-27* genotypes and alleles in RPL and control group

**SNP**	**Allele frequency**		**Genotype frequency**		
**-964A>G**	**A Allele**	**G Allele**	**AA**	**AG**	**GG**
RPL group	194 (64.7)*	106(35.3)	60 (40)**	74 (49.3)	16 (10.7)
Control group	211 (70.3)	89(29.7)	73 (48.7)	65 (43.3)	12 (8)

Data are presented as n (%).

RPL= Recurrent pregnancy loss

* p=0.19

** p=0.23

## Discussion

It has been postulated that IL-27 promotes Th1 response and suppress Th17 differentiation (10, 19). Most of the recent works focused on a role for this cytokine in inducing IL-10 production (20). IL-10 is a potent anti-inflammatory cytokine that restrains inflammation in a variety of contexts. Previously Hadinedoushan *et al* reported the elevation of Th1 cytokines in women with recurrent miscarriage history compared to normal pregnant women, and IL-10 as an important cytokine in the maintenance of pregnancy (21). An imbalance between Th1 and Th2 cells and increased numbers of Th17 cells in RPL were observed by Lee *et al* (22). It is believed that Th1 and Th17 cells are actively involved in the pro-inflammatory immune response at the maternal-fetal junction at the time of implantation which could subsequently lead to the development of RPL.(8). 

This study tried to investigate the probable influence of genotypic and allelic variants in IL-27p28 on susceptibility to RPL in Iranian women. Our results showed that the frequency of allele A was 64.7% in the RPL group and 70.3% in the control group and there was no difference in frequency of allele A in -964 A>G between the two groups. The genotype AG (49.3%) in the RPL group and genotype AA (48.7%) in the control group had the highest frequency. The genotype GG in both case (10.7%) and control (8%) groups had the lowest frequency. 

Chae *et al* studied IL-27-964 A>G polymorphism in asthma patients and healthy control in a Korean population. The AA genotype frequency was 48.6% in control and 60.8% in asthma patients. The GG frequency both in control (8.6%) and patients (6.6%) was the lowest frequency (16). 

The association of this polymorphism with chronic hepatitis B virus infection was determined in Chinese patients and healthy individuals. The results showed that the AA genotype frequency was 56.2% in patients with chronic hepatitis B, 40.4% in recovered individuals and 42.1% in healthy subjects. The GG genotype had the lowest frequency in all three groups (23). The SNP was also evaluated in patients with type 1 diabetes mellitus and healthy control subjects in Brazil. The AA genotype frequency was 34% in patients and 33.1% in controls (24). Our results are consistent with the findings of other investigators in Asian countries, however; it disagrees with the same study that was done in Brazil. The difference in the race might explain this discrepancy. 

We found that genotype frequencies of -964 A>G in *IL-28p28* gene were not significantly different in the control and RPL groups. Our data suggest that *IL-27p28* gene polymorphism may not be the important contributor to RPL. To our knowledge, this is the first report to attempt an evaluation of the association between the SNP of *IL-27p28* gene and RPL susceptibility. The -964 A>G variant of *IL-27p28* gene have been shown to be associated with susceptibility to some disorders. Li *et al* studied *IL-27* polymorphisms in inflammatory bowel diseases and healthy controls in Korean population. The genotypes frequencies of the -964 A>G polymorphism in the patients were significantly different from those of the healthy control group (17). The association of this polymorphism with susceptibility to asthma has been reported (16). 

Huang *et al* studied association of *IL-27* gene polymorphisms with chronic obstructive pulmonary disease in a Chinese population. The results showed that there were significant difference in the genotype and allele distribution of -964 A>G polymorphism of the *IL-27p28* gene between case and control groups. When compared with the control group, samples with AG genotype of *IL-27* -964 A>G had 2.22-fold decreased risk of disease (25). The mentioned diseases are associated with Th2 immune response. IL-27 can down regulate Th2 type cytokines and the presence of this polymorphism could influence the Th shift. There are some reports that fail to show the association between *IL-27p28* gene polymorphisms and diseases. 

Zhao *et al* investigated the association of *IL-27* -964 A>G polymorphism with immune thrombocytopenia. They did not find significant difference in genotype and allele’s distribution between patients and the healthy controls (26). A study on type 1 diabetes and controls showed that the frequency of the alleles and genotypes of *IL-27p28* did not differ between patients and controls and also, there was no association between this polymorphism and gender, ethnicity, age at diagnosis and extra pancreatic autoantibodies (24). The discrepancy between our results and other findings could be due to the difference in populations, the subjects and sample sizes. Further studies in different population are needed to identify the association between *IL-27* gene polymorphism and RPL. 

## Conclusion

In conclusion, our results suggest that the *IL-27p28* (-964 A/G) gene polymorphism is not a risk factor for RPL in Iranian women.

## Note

***This article extracted from M.Sc. thesis. (Zeinab Nematollahi)***
